# Omega-3 Polyunsaturated Fatty Acids Enhance Neuronal Differentiation in Cultured Rat Neural Stem Cells

**DOI:** 10.1155/2013/490476

**Published:** 2013-01-14

**Authors:** Masanori Katakura, Michio Hashimoto, Toshiyuki Okui, Hossain Md Shahdat, Kentaro Matsuzaki, Osamu Shido

**Affiliations:** Department of Environmental Physiology, Faculty of Medicine, Shimane University, Shimane Izumo, 693-8501, Japan

## Abstract

Polyunsaturated fatty acids (PUFAs) can induce neurogenesis and recovery from brain diseases. However, the exact mechanisms of the beneficial effects of PUFAs have not been conclusively described. We recently reported that docosahexaenoic acid (DHA) induced neuronal differentiation by decreasing Hes1 expression and increasing p27^kip1^ expression, which causes cell cycle arrest in neural stem cells (NSCs). In the present study, we examined the effect of eicosapentaenoic acid (EPA) and arachidonic acid (AA) on differentiation, expression of basic helix-loop-helix transcription factors (Hes1, Hes6, and NeuroD), and the cell cycle of cultured NSCs. EPA also increased mRNA levels of Hes1, an inhibitor of neuronal differentiation, Hes6, an inhibitor of Hes1, NeuroD, and Map2 mRNA and Tuj-1-positive cells (a neuronal marker), indicating that EPA induced neuronal differentiation. EPA increased the mRNA levels of p21^cip1^ and p27^kip1^, a cyclin-dependent kinase inhibitor, which indicated that EPA induced cell cycle arrest. Treatment with AA decreased Hes1 mRNA but did not affect NeuroD and Map2 mRNA levels. Furthermore, AA did not affect the number of Tuj-1-positive cells or cell cycle progression. These results indicated that EPA could be involved in neuronal differentiation by mechanisms alternative to those of DHA, whereas AA did not affect neuronal differentiation in NSCs.

## 1. Introduction

Polyunsaturated fatty acids (PUFAs) are critical for the developing brain and are classified into omega-3 PUFAs, such as eicosapentaenoic acid (EPA) and docosahexaenoic acid (DHA), and omega-6 PUFAs, such as arachidonic acid (AA). Only low levels of many PUFAs are synthesized from their respective shorter-chain precursors in mammals; thus, they need to be obtained from dietary sources. Dysregulation of fatty acid and phospholipid metabolism can induce a wide range of psychiatric, neurological, and developmental disorders in adults [1].

The enhancement of neurogenesis is an important tool to treat brain disorders and has been shown to ameliorate or prevent mental illnesses [2], cholinergic denervation [3], and neurodegenerative diseases [4]. Omega-3 PUFAs reportedly enhanced neurogenesis in adult rat hippocampi [5], brain tissue of lobsters [6], and in fat-1 transgenic mice [7]. Bertrand et al. [8] show that cortical development is disrupted by feeding omega-3 deficient diets in embryonic rats. AA has also been shown to enhance neurogenesis in rat hippocampi [9], and AA enhance proliferation and astrogenesis of fetal rat neuronal stem/progenitor cells (NSCs) [10]. However, the exact mechanisms of the beneficial effect of PUFAs on neurogenesis have not been elucidated.

Neurogenesis comprises the proliferation and differentiation of NSCs, which involves separate mechanisms [11]; therefore, in the present study, we focused on the differentiation of NSCs. We previously reported that DHA decreased Hes1 expression in NSCs [12], and Hes1, a repressor type of basic helix-loop-helix (bHLH) transcription factor, is essential for the maintenance and proliferation of NSCs [13], and their expression maintains the NSCs during embryogenesis [14]. Activator-type bHLH transcription factors such as Hes6, neurogenin, Mash1, and NeuroD enhanced the expression of MAP2, a neuron specific protein, and induced neuronal differentiation. Crosstalk between these two types of bHLH transcription factors allows some NSCs to undergo differentiation and maintain an NSC state.

Regulation of the cell cycle plays an important role in cell proliferation, differentiation, and apoptosis of NSCs. Neuronal differentiation is highly coordinated by numerous factors, including transcription factors, trophic factors, and cell cycle regulators [15, 16]. Prior to differentiation, cells are arrested at the G1/S phase and enter the G0 phase without passing the cell cycle restriction point. Deferoxamine, a G1/S phase blocker, promotes neuronal differentiation of NSCs [17]. We previously observed that DHA increased p27^kip1^ expression and induced cell cycle arrest [12], indicating the importance of cell cycle regulation for differentiation of NSCs. In this study, we evaluated the effects of EPA and AA in comparison with the effects of DHA on bHLH transcription factors and cell cycle regulation under differentiation conditions using cultured NSCs.

## 2. Materials and Methods

### 2.1. Animals

Pregnant female rats (Wistar; Clea Japan, Inc., Tokyo, Japan) at embryonic day (E) 14.5 were used. All experiments were carried out in accordance with the Guidelines for Animal Experimentation of the Center for Integrated Research in Science, Shimane University (Shimane, Japan), and were approved by the Animal Care and Use Committee of the same institution and the Guiding Principles for the Care and Use of Animals in the Field of Physiological Science of the Physiological Society of Japan. A minimum number of anesthetized rats were used for the collection of embryonic NSCs.

### 2.2. Culture of Embryonic NSCs

NSCs were cultured by the neurosphere method as described previously [12, 18]. The cortices were mechanically disrupted into single cells by repeated pipetting in a serum-free conditioned medium (N_2_ medium) containing DMEM/F12 (1 : 1), 0.6% (w/v) glucose, 0.1125% (w/v) sodium bicarbonate, 2 mM L-glutamine, 5 mM HEPES, 100 *μ*g/mL human apotransferrin, 20 nM progesterone, 30 nM sodium selenite, 60 *μ*M putrescine, and 25 *μ*g/mL insulin. The dissociated cells were cultured in 6 cm dishes at a density of 1 × 10^5^ cells/mL in N_2_ medium with 20 ng/mL basic fibroblast growth factor (bFGF) and 2 *μ*g/mL heparin in a humidified 5% CO_2_/95% air incubator at 37°C. Within 3–5 days, the cells grew as free-floating neurospheres that were then collected by centrifugation, mechanically dissociated by pipetting, and passaged. Three hours after culturing in N_2_ medium without bFGF, the plated cells mostly included nestin-positive cells (a neural stem cell marker; 95.3 ± 1.7%; *n* = 6), prominin-1 (CD133)-positive cells (a neural stem cell marker; 97.24 ± 0.2%; *n* = 6), and few neuron-specific class III beta-tubulin-(Tuj-1-) and glial fibrillary acidic protein-(GFAP-) positive cells (<3%).

### 2.3. Differentiation of NSCs

After the second passage, neurospheres were mechanically dissociated, and 2 × 10^5^ cells were plated onto poly-*L*-ornithine-coated (15 *μ*g/mL; Sigma-Aldrich, St. Louis, MO, USA) 24-well plates containing N_2_ medium without bFGF and heparin. The cultures were then treated with PUFAs (DHA, EPA, or AA; 1.0 *μ*M; Sigma-Aldrich) that were dissolved in N_2_ medium containing 1.0% fatty acid-free bovine serum albumin (BSA; Sigma-Aldrich) at a final concentration of 0.01%. BSA was used as the vehicle control in this experiment, and the culture medium was changed every other day.

### 2.4. Cell Viability Assay

NSCs were seeded onto poly-*L*-ornithine-coated 24-well plates at a density of 2 × 10^5^ cells/well in N_2_ medium with or without PUFAs. The methyl thiazol tetrazolium assay (MTT; 3-(4,5-dimethylthiazol-2-yl) 2,5-diphenyltetrazolium bromide; Dojindo Laboratories, Kumamoto, Japan) was conducted to measure cell viability. The cells were incubated with 0.25 mg/mL of MTT at 37°C for 4 h; the reaction was terminated by the addition of 20% sodium dodecyl sulfate/50% dimethylformamide; the plates were then gently shaken at room temperature for 12 h before extracting the MTT formazan product which was quantified using a microplate reader at an absorbance of 550 nm. The data are expressed as percentages of control.

### 2.5. Immunofluorescence Staining

Cultured cells were fixed with 4% paraformaldehyde for 30 min at room temperature, washed with 0.1 M Tris-buffered solution (TBS; pH 7.5), blocked with 3% normal goat serum (Dako Cytomation, Carpinteria, CA, USA) in TBS containing 0.3% Triton X-100 at room temperature for 60 min, and incubated overnight with primary antibodies (mouse anti-Tuj-1, 1 : 1000, R&D Systems, Inc., Minneapolis, MN, USA, and rabbit anti-GFAP, 1 : 1000, Sigma) at 4°C. The cells were then washed with TBS and incubated with Alexa Fluor 488-conjugated secondary antibody (1 : 1000; Invitrogen Corp., Carlsbad, CA, USA) at room temperature for 60 min. To visualize the nuclei, the cells were counterstained with 2 *μ*g/mL of propidium iodide (PI; Dojindo Laboratories). Finally, the cells were mounted with 80% of glycerol and visualized under a fluorescent laser microscope (CLMS FV300; Olympus Corp., Tokyo, Japan). The images were processed using Image J software (National Institute of Health, Bethesda, MD, USA). The number of Tuj-1-positive, GFAP-positive, and total cells was counted in each of the seven random fields per well.

### 2.6. Real-Time PCR

The NSCs were allowed to differentiate for day 1 to 4 days in the differentiation medium in the presence of 1 *μ*M PUFAs. Total RNA was isolated using Isogen reagent (Wako Pure Chemical Industries, Ltd., Tokyo, Japan), and then cDNA was synthesized with the QuantiTect Reverse Transcription Kit (Qiagen GmbH, Hilden, Germany) and amplified using the ABI prism 7000 sequence detection system (Applied Biosystems Inc., Foster City, CA, USA). Real-time polymerase chain reaction PCR was carried out with the QuantiTect SYBR Green PCR Kit (Qiagen). The primer sequences are listed in Table 1. The specificity of PCR products was confirmed by both melting curve analysis and agarose gel electrophoresis. In the preliminary experiment, we determined the amplification efficiencies of all genes, which were similar. The PCR conditions were as follows: initial activation at 95°C for 15 min, then 40 amplification cycles of denaturation at 94°C for 15 s, annealing at 58°C for 30 s, and extension at 72°C for 30 s. The relative changes in gene expression levels were determined by the 2^−ΔΔCt^ method described in User Bulletin #2 of the ABI prism 7000 sequence detection system.

### 2.7. Cell Cycle Analysis

Cell cycle analysis was performed using a BrdU Flow Kit (Becton Dickinson and Company, SanDiego, CA, USA) following to manufacturer's instruction. After 11 h of incubation, 5-bromo-2′-deoxyuridine (BrdU; 10 *μ*M) was added to the culture medium, and the cells were incubated for an additional 1 h. Cells were harvested stained for incorporated BrdU by fluorescein isothiocyanate-(FITC-) conjugated anti-BrdU antibody and for total DNA by 7-amino-actinomycin D (7-AAD). Cells were analyzed by fluorescence-activated cell sorting (FACS) using Becton Dickinson FACSCalibur flow cytometer equipped with a 15 mW, 488 nm, air-cooled argon ion laser for excitation of FITC (FL1), and 7-AAD (FL3). FL1 height (FL1-H) signals were collected after logarithmic amplification, while height signals for forward light scatter (FSC-H) and side light scatter (SSC-H) and FL3-A area signals were collected after linear amplification. Samples were acquired and analyzed using CELLQuest 6.1.0 software (Becton Dickinson). Gates for the quantitative cell cycle analysis of populations that have been stained for incorporated BrdU and total DNA levels and the percentage of cells in the G0/G1, S, and G2/M phases were determined.

### 2.8. Statistical Analysis

Statistical analysis was carried out by one-way analysis of variance (ANOVA), and the results are expressed as the means ± standard error (SE). One-way ANOVA followed by the Dunnett's test was used for comparisons with the control group. *P* < 0.05 was considered statistically significant.

## 3. Results

### 3.1. Effects of PUFAs on Neuronal or Glial Differentiation and Cell Viability of NSCs

**Figure 1:**
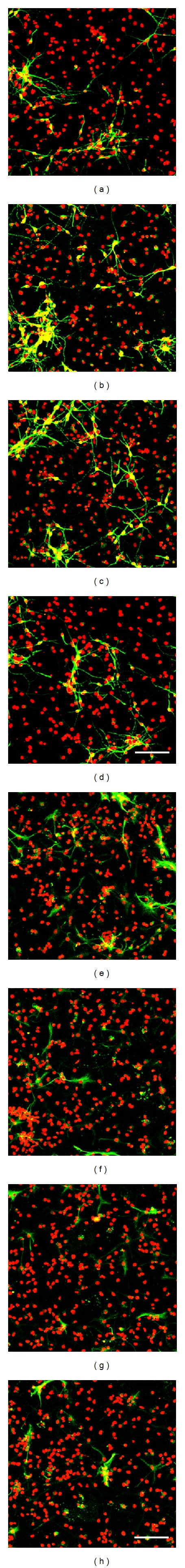
Immunofluorescence images of differentiated neural stem cells (NSCs) stained with Tuj-1 (a neuron marker, (a)–(d)) or GFAP (an astrocyte marker, (e)–(h)) and PI (nuclei) in control (0.01% BSA treated, (a) and (e)), DHA (1 *μ*M, (b) and (f)), EPA (1 *μ*M, (c) and (g)), and AA (1 *μ*M, (d) and (h)) groups on treatment day 4. White bar indicates 10 *μ*m.

**Figure 2:**
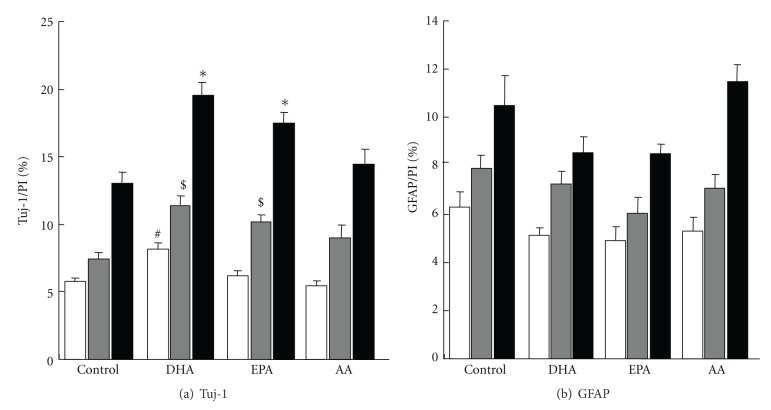
Quantification of Tuj-1 (a) or GFAP (b) positive cells in control, DHA, EPA, and AA groups after 2 (white), 4 (gray), and 7 (black) days. Values are presented as means ± SE for five experiments. Statistical analyses were performed by ANOVA followed by Dunnett's test. ^#,$,∗^ *P* < 0.05 versus control group.

Cell viability was analyzed using MTT assay by treating with 1 *μ*M of DHA, EPA, AA, or 0.01% BSA as a control for 4 days. No change in cell viability was detected in any PUFA-treated NSCs (data not shown), indicating that 1 *μ*M PUFA was not toxic to NSCs. On day 4, after differentiation, neuronal cells were identified by staining with anti-Tuj-1 antibody in cells treated with 1 *μ*M of DHA, EPA, or AA (green, Figures 1(a)–1(d)), whereas astrocytes were identified by staining with anti-GFAP antibody (green, Figures 1(e)–1(h)). Tuj-1-positive cells increased significantly after 4 and 7 days of DHA and EPA treatment, while no difference was observed in AA-treated NSCs (Figure 2(a)). These data indicated that omga-3 PUFAs, but not omega-6 PUFAs, increased neuronal differentiation of NSCs. However, the number of GFAP-positive cells was not affected by treatment with any PUFA (Figure 2(b)).

### 3.2. PUFA Effects on mRNA Expression of bHLH Transcription Factors

**Figure 3:**
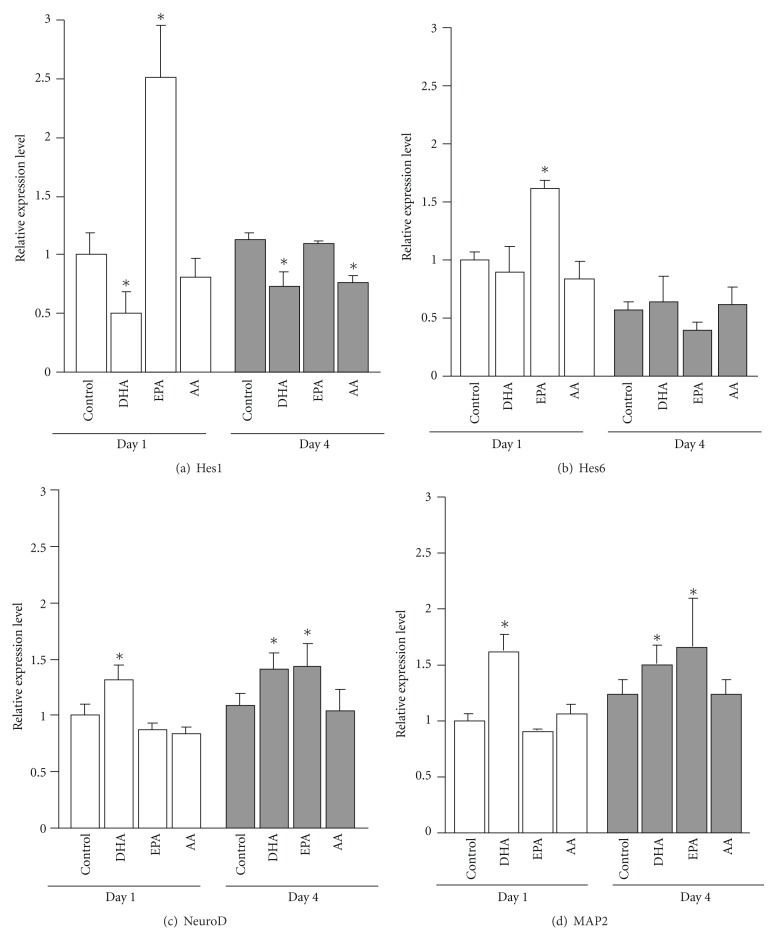
Effects of PUFA on mRNA expression levels of bHLH transcription factors. The values are expressed as the mean ± SE of the fold increase in the ratio of each gene/GAPDH, with the value of the control group (Day 1) taken as 1.0. Statistical analysis was performed by ANOVA followed by Dunnett's test. *P < 0.05 versus control group.

Hes1 mRNA levels were decreased by DHA treatment on day 1 day and 4 day, whereas EPA treatment increased Hes1 mRNA expression by 2.5-fold on day 1. AA treatment on day 4 significantly decreased Hes1 mRNA expression (Figure 3(a)). Hes6, an inhibitor of Hes1, was also significantly increased by EPA treatment on day 1, but not on day 4 (Figure 3(b)). DHA and EPA treatment significantly increased NeuroD mRNA expression, but AA did not have any effect (Figure 3(c)).  Figure 3(d) shows that the expression levels of Map2 mRNA significantly increased with DHA and EPA treatment, reflecting the change in Hes1, Hes6, and NeuroD expression levels in Tuj-1-positive cells.

### 3.3. Effects of PUFAs on the Cell Cycle in NSCs

**Figure 4:**
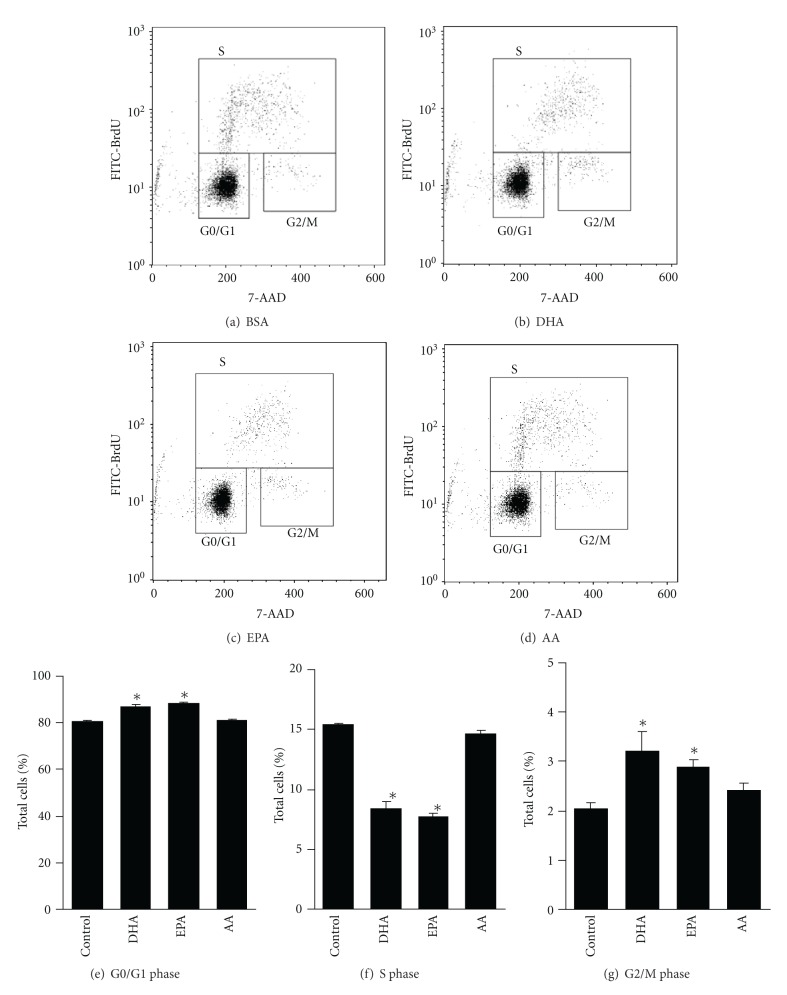
Cell cycle analysis in NSCs after treatment with or without PUFAs. Incorporation of BrdU and total DNA content (7-amino-actinomycin D, 7-AAD) in cells treated with BSA (a), 1 *μ*M DHA (b), EPA (c), or AA (d) were measured by flow cytometry. The cell ycle phase was determined by counting each gate. The percentage of cells distributed in each phase of the cell cycle is shown: G0/G1 phase (e), S phase (f), and G2/M phase (g). Values are means ± SE of three independent experiments. *P < 0.05 versus control group. FITC-BrdU is fluorescein isothiocyanate-conjugated anti BrdU antibody.

**Figure 5:**
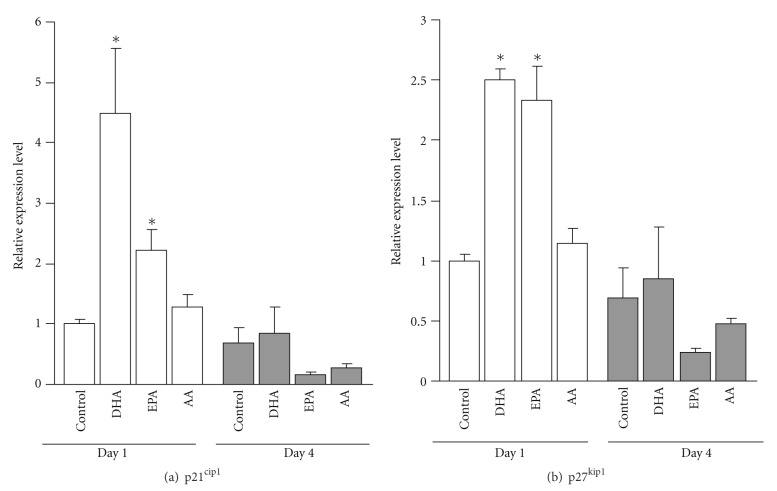
Effects of PUFAs on mRNA expression levels of p21^cip1^ and p27^kip1^. The values are expressed as the means ± SE of the fold increase in the ratio of each gene versus GAPDH, with the value of the control group (day 1) taken as 1.0. Statistical analysis was performed by ANOVA followed by Dunnett's test. *P < 0.05 versus control group.

Next, we analyzed BrdU incorporation and total DNA content (7-AAD) in differentiating NSCs treated with DHA, EPA, AA, or 0.01% BSA.  Figure 4 shows a 7-AAD versus BrdU (FITC-/anti-BrdU) dot plot. Proliferating cells incorporated BrdU into their DNA and increased the FITC signal intensities. Cell cycle analysis 12 h after DHA and EPA treatment revealed a significant increase in the percentage of G0/G1 phase cells (control 80.8 ± 0.1%; DHA 87.0 ± 0.8%; EPA 88.5 ± 0.2%) and a significant decrease in the percentage of S phase cells (control 15.4 ± 0.1%; DHA 8.4 ± 0.5%; EPA 7.8 ± 0.3%). On the other hand, following AA treatment, 81.2 ± 0.2% of cells were in the G0/G1 phase, 14.6 ± 0.3% in the S phase, and 2.4 ± 0.2% in the G2/M phase. To confirm these results, mRNA expression levels of p21^cip1^ and p27^kip1^ (cyclin-dependent kinase inhibitors) were determined in PUFA-treated NSCs. p21^cip1^ mRNA levels in the NSCs treated with DHA and EPA were 4.5- and 2.2-fold higher than in controls, respectively (Figure 5(a)), and p27^kip1^ mRNA levels were 2.5- and 2.3-fold higher than in controls, respectively (Figure 5(b)).

## 4. Discussion

It was observed that EPA treatment induced neuronal differentiation in cultured NSCs and increased mRNA levels of Hes1, an inhibitory factor of neuronal differentiation, and Hes6, an inhibitory factor of Hes1. EPA also caused NeuroD and Map2 mRNA levels to increase in Tuj-1-positive cells. EPA arrested the cell cycle of NSCs through increased p21^cip1^ and p27^kip1^ levels in the N_2_ medium without bFGF. AA treatment decreased Hes1 mRNA levels, but not NeuroD and Map2 mRNA levels. Furthermore, AA did not affect the number of Tuj-1-positive cells or cell cycle progression. These results indicated that omega-3 PUFAs, but not AA, increased neuronal differentiation.

Omega-3 PUFAs enhanced neurogenesis in the adult rat hippocampus [5], embryonic rat brain [6], lobster brain [7], and in fat-1 transgenic mice [8]. Kan et al. [19] reported that treatment with both DHA and AA induced neuronal differentiation of bone marrow-derived mesenchymal stem cells compared with DHA or AA only, indicating that DHA and AA may have a synergistic effect on neurogenesis. In the present study, AA had no effect on NSC differentiation. These data were consistent with a previous study in which AA had no effect on neuronal differentiation of neurogenic and gliogenic NSCs [10]. However, it has been reported that AA promoted neurogenesis in 4-5-week-old juvenile rats [9]. These conflicting results could be due to AA metabolites that might influence neurogenesis. Uchida et al. [20] reported that prostaglandin E_2_, synthesized by cyclooxygenase from AA, increased neurogenesis in the subgranular zone of the dentate gyrus in the hippocampus of adult rats. Manev et al. [21] observed that 5′-lipoxygenase may also participate in neurogenesis; however, further experiments are necessary to clarify these points. Sakayori et al. [10] reported that 0.01 *μ*M DHA increased the Tuj-1-positive cells from tertiary neurospheres (gliogenic NSCs) but had no detectable effect on primary neurospheres (neurogenic NSCs). In this study, we used secondary neurospheres and did not stain the cells with GFAP before differentiation. These results indicated that NSCs in the present experiment were neurogenic NSCs. In contrast to our results, DHA did not affect the number of Tuj-1-positive cells in neurogenic NSCs [10]. Sakayori et al. [10] collected the NSCs from E16.5 fetal rat cortices and cultured them in a medium supplemented with bFGF, epidermal growth factor (EGF), and heparin. In contrast, we used NSCs from E14.5 fetal rat cortices and cultured them in the medium with bFGF and heparin, but without EGF; therefore, the difference between the results of the two studies can be explained by the differences in culture media. It has been reported that there is a differential response to EGF and bFGF (both alone and in combination) depending on the gestational age at isolation [22].

Hes1 inhibits neuronal differentiation in NSCs by inhibiting pre neuronal factors and inducing proliferation. In the present study, EPA increased Hes1 mRNA expression, Map2 mRNA expressions and the number of Tuj-1-positive cells because Hes6 acts in a positive feedback loop in neurogenesis by competing with Hes1 activation and promoting proteolytic degradation of Hes1 [23, 24]. In this study, EPA increased Hes6 mRNA levels, suggesting that it inhibited Hes1-induced NSC proliferation. Although Hes1 protein levels were not measured in the present study, mRNA levels of the Hes1 target genes, NeuroD, and p21^cip^ were decreased, suggesting that the Hes1 protein may have decreased under this experimental condition. This was an EPA specific effect because DHA and AA did not affect Hes6 expression. 

The *Notch-Hes1* pathway may contribute to the adequate proliferation of sensory precursor cells via transcriptional downregulation of p27^kip1^ expression by Hes1, which directly binds to the p27^kip1^ promoter region [25, 26]. Hes1 activation may also repress p21^cip1^ transcription in pheochromocytoma-(PC-) 12 cells [27]. Therefore, Hes1 inhibition may affect cell cycle regulation. Hes6 expression appears to activate p21^cip1^ transcription, suppresses cell proliferation [28], and may also affect the cell cycle. We previously demonstrated that DHA inhibited Hes1 expression and arrested the cell cycle in NSCs [12], and the present study demonstrated that DHA and EPA increased G0/G1 phase cells, decreased S phase cells, and increased p21^cip1^ and p27^kip1^ mRNA levels, consistent with Hes1 and/or Hes6 regulation by DHA and EPA. We should mention that DHA- and EPA-induced cell cycle arrest was observed in N_2_ medium without bFGF supplementation. 

In the present study, we found that DHA and EPA enhanced neuronal differentiation in cultured NSCs. However, their target effector molecules are different. Our results indicated that EPA itself acts as an enhancer for neuronal differentiation, and EPA did not serve solely as a precursor for DHA. Previous reports have shown different effects of DHA and EPA on neuronal function [29]. In contrast to DHA, it is not clear whether EPA enhanced neuronal differentiation in NSCs in vivo. On comparing with the presence of DHA, EPA is present at a very low concentration within brain phospholipids. However, single and long-term administration of EPA significantly increased its concentration in the rat brain [30, 31] and EPA readily crossed the blood-brain barrier, as did DHA, in an in situ cerebral perfusion study [32]. These reports indicated that EPA was incorporated into brain tissue and may have singularly increased neuronal differentiation in NSCs in vivo; therefore, EPA may be useful for the prevention and treatment of neurological disorders by inducing neurogenesis.

## 5. Conclusion

**Figure 6:**
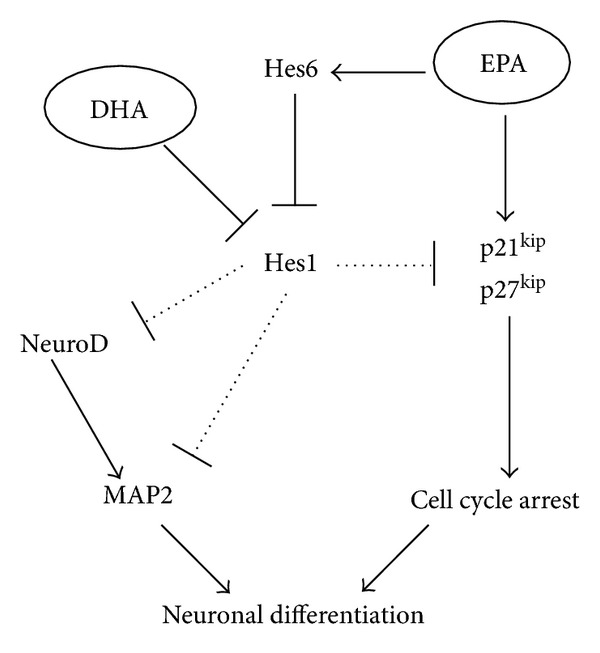
Mechanisms of omega-3PUFA-induced neuronal differentiation in NSCs.

The mechanisms of *ω*-3PUFA-induced neuronal differentiation in NSCs are shown in Figure 6. DHA decreased Hes1 expression levels and EPA increased Hes6 expression levels, which led to a decreased Hes1 activity. Decreased Hes1 activity increased NeuroD, Map2, p21^cip1^, and p27^kip1^ expression levels. These mechanisms promoted neuronal differentiation in NSCs.

## Figures and Tables

**Table 1:** List of primers for real-time PCR.

Gene	Forward sequence	Reverse sequence
Hes1	TTCCTCCCATTGGCTGAAAG	CCAGCTCCAGATCCAGTGTGAT
Hes6	TCCTTAGGCATCCTGACCAC	TGGGCTATCTCCACCTCATC
NeuroD	AAGACGCATGAAGGCCAATG	GCCAAGCGCAGTGTCTCTATCT
Map2	GTTTACATTGTTCAGGACCTCATGG	TCGGTAAGAAAGCCAGTGTGGT
p27^kip1^	GGCGAAGAGAACAGAAGAAAATG	GGGCGTCTGCTCCACAGT
p21^cip1^	CAAAGTATGCCGTCGTCTGTTC	CATGAGCGCATCGCAATC
Gapdh	ATCTTCTTGTGCAGTGCCAGC	CCTTGACTGTGCCGTTGAACT

Hes: hairy and enhancer of split; Map2: microtubule-associated protein 2; Gapdh: glyceraldehydes-3-phosphate dehydrogenase.
